# A pooled analysis evaluating prognostic significance of Residual Cancer Burden in invasive lobular breast cancer

**DOI:** 10.1038/s41523-025-00720-3

**Published:** 2025-02-13

**Authors:** Rita A. Mukhtar, Soumya Gottipati, Christina Yau, Sara López-Tarruella, Helena Earl, Larry Hayward, Louise Hiller, Marie Osdoit, Marieke van der Noordaa, Diane de Croze, Anne-Sophie Hamy, Marick Laé, Fabien Reyal, Gabe S. Sonke, Tessa G. Steenbruggen, Maartje van Seijen, Jelle Wesseling, Miguel Martín, Maria del Monte-Millán, Judy C. Boughey, Matthew P. Goetz, Tanya Hoskin, Vicente Valero, Stephen B. Edge, Jean E. Abraham, John M. S. Bartlett, Carlos Caldas, Janet Dunn, Elena Provenzano, Stephen-John Sammut, Jeremy S. Thomas, Ashley Graham, Peter Hall, Lorna Mackintosh, Fang Fan, Andrew K. Godwin, Kelsey Schwensen, Priyanka Sharma, Angela M. DeMichele, Kimberly Cole, Lajos Pusztai, Mi-Ok Kim, Laura J van ’t Veer, David Cameron, Laura J. Esserman, W. Fraser Symmans

**Affiliations:** 1 University of California San Francisco, San Francisco, USA; 2 Instituto de Investigación Sanitaria Gregorio Marañón Madrid, Madrid, Spain; 3 University of Cambridge, Cambridge, UK; 4 Western General Hospital, Edinburgh, UK; 5 University of Warwick, Coventry, UK; 6 Institut Curie, Paris, France; 7 Université de Rouen, Rouen, France; 8 Netherlands Cancer Institute, Amsterdam, The Netherlands; 9 Mayo Clinic, Rochester, USA; 10 University of Texas MD Anderson Cancer Center, Houston, USA; 11 Roswell Park Cancer Institute, Buffalo, USA; 12 Ontario Institute for Cancer Research, Toronto, Canada; 13 Edinburgh Cancer Research Centre, Edinburgh, UK; 14 University of Toronto, Toronto, Canada; 15 University of Kansas Medical Center, Kansas City, USA; 16 University of Pennsylvania, Philadelphia, USA; 17 Yale University, New Haven, USA

**Keywords:** Breast cancer, Prognostic markers

## Abstract

Residual Cancer Burden (RCB) after neoadjuvant chemotherapy (NAC) is validated to predict event-free survival (EFS) in breast cancer but has not been studied for invasive lobular carcinoma (ILC). We studied patient-level data from a pooled cohort across 12 institutions. Associations between RCB index, class, and EFS were assessed in ILC and non-ILC with mixed effect Cox models and multivariable analyses. Recursive partitioning was used in an exploratory model to stratify prognosis by RCB components. Of 5106 patients, the diagnosis was ILC in 216 and non-ILC in 4890. Increased RCB index was associated with worse EFS in both ILC and non-ILC (*p* = 0.002 and *p* < 0.001, respectively) and remained prognostic when stratified by receptor subtype and adjusted for age, grade, T category, and nodal status. Recursive partitioning demonstrated residual invasive cancer cellularity as most prognostic in ILC. These results underscore the utility of RCB for evaluating NAC response in those with ILC.

## Introduction

Neoadjuvant chemotherapy (NAC) for breast cancer increases rates of successful breast-conserving surgery, and importantly, can provide a means of evaluating response to treatment that provides prognostic information and guides adjuvant therapy^1–3^. The absence of residual invasive disease after NAC, termed pathologic complete response (pCR), is associated with significantly improved event free survival (EFS)^4^. While pCR is a binary outcome, quantifying the degree of tumor response to NAC is possible with the Residual Cancer Burden (RCB) method. The RCB evaluation is performed on post-treatment surgical specimens, and includes an assessment of primary tumor bed size, overall cancer cellularity, percentage of carcinoma cells that are in situ, number of positive lymph nodes, and the size of the largest lymph node metastasis^5,6^. As both a continuous index and categorical measurement (RCB classes 0–III, where RCB 0 indicates pCR), RCB has been validated as a robust predictor of EFS for all receptor subtypes of breast cancer^7^.

However, whether RCB is associated with EFS to the same extent among patients with differing histologic subtypes, such as invasive lobular carcinoma (ILC), is less well understood. The second most common histologic subtype of breast cancer, ILC is a unique tumor type that is largely hormone receptor (HR) positive and human epidermal growth factor-2 (HER2) negative^8^. The characteristic feature of ILC is the absence of the adhesion protein E-cadherin, which leads to a diffuse growth pattern with single file lines of tumor cells. Due to this diffuse growth pattern, standard imaging tools have lower sensitivity for ILC, and patients with ILC are diagnosed at more advanced stages than those with the more common invasive ductal carcinoma (IDC)^9–12^.

Another challenge in the management of ILC is the very low rate of pCR observed in this subtype. While a subset of ILC has more molecularly aggressive features (e.g., pleomorphic ILC, or the uncommon triple negative or HER2 amplified cases) and may achieve pCR more often, multiple studies demonstrate significantly lower pCR rates in ILC compared to IDC^13–15^. These low rates make pCR a less useful clinical endpoint for both clinical trials and clinical management of patients with this tumor type, which represents 10–15% of all diagnosed breast cancer cases. For this reason, the RCB method, which allows for quantification of partial responses to NAC, may be particularly useful for those with ILC who receive pre-operative chemotherapy.

In this study, we aimed to validate the RCB method for predicting event free survival (EFS) in patients with ILC who received NAC. We utilized an international, multicenter, pooled cohort in which we previously reported associations between RCB and EFS by tumor receptor subtype across all histologic subtypes, and now evaluate ILC cases specifically compared to non-ILC cases^7^. Our primary study question was whether or not RCB index and class are associated with EFS in patients with ILC. Additionally, we explored which of the individual components of RCB was most predictive of outcomes in those with ILC.

## Methods

### Data source

We studied patient-level data (RCB, histologic subtype, and EFS) from a previously described pooled cohort from 12 institutions in the United States and Europe^7^. Patients included were age ≥18 years with stage I-III invasive breast cancer and underwent neoadjuvant chemotherapy prior to surgery. Neoadjuvant regimens varied across studies, with 2 studies using investigational treatments and 10 studies using standard of care regimens. While treatment regimens were tailored to receptor subtype (HR and HER2 status), tumor histology (ILC versus non-ILC) was not utilized for treatment selection in any cohorts. Accordingly, ILC and non-ILC patients received the same therapy within each study. All studies followed standard of care recommendations for adjuvant therapy, with one study enrolling patients prior to the approval of trastuzumab for HER2 amplified tumors. De-identified data from the 12 institutions (four clinical trials and eight clinical cohorts) were collated in November 2019 after obtaining institutional review board approvals. All studies were conducted in accordance with the Declaration of Helsinki, with written informed consent obtained for 7 contributing cohorts (including all clinical trials), and 5 cohorts having this requirement waived by the approving review board due to no patient contact or intervention, as described in Supplementary Table 1. Data are presented adhering to recommendations from Strengthening the Reporting of Observational Studies in Epidemiology (STROBE) guidelines^16^.

RCB was evaluated by breast cancer pathologists at each institution, in a prospective fashion for five cohorts and retrospectively for seven cohorts. Standard RCB assessment training was provided either one-on-one or via a centrally produced training video. Calculated from post-neoadjuvant treatment surgical specimens, RCB includes tumor bed dimensions, residual tumor cellularity, the percentage of in situ carcinoma, and quantification of residual tumor burden in lymph nodes. The estimates of overall cancer cellularity and its in situ component are algebraically combined to estimate the invasive cancer cellularity as a percent of the area of tumor bed^5^. RCB is reported as a continuous index or grouped into RCB classes (0, I, II, III) corresponding with no residual invasive disease (RCB 0) or progressively increasing residual disease burden, with RCB-I indicating a score ≥0–1.36, RCB II indicating a score 1.37–3.28, and RCB III indicating a score >3.28.

The histologic diagnosis of ILC was made according to local institutional protocols. We surveyed participating sites to determine whether immunohistochemical staining to aid in diagnosis of ILC was routinely performed or in select cases only. Of 12 participating institutions, 11 (91.7%) reported using selective E-cadherin staining for ILC diagnosis only when morphologic status was unclear, with one institution reporting obligatory use of E-cadherin staining for all diagnoses of ILC. Of the institutions using selective staining, 7 sites estimated that E-cadherin staining was used in 25–50% of cases diagnosed as ILC, 2 reported E-cadherin staining in 50–75% of ILC cases, and 2 reported E-cadherin staining in >75% of cases. There were 5161 patients across the 12 sites. Of these, histology reported was ductal or mixed ductal in 4790, lobular in 216, other in 100, and unknown in 55. Among the mixed ductal cases, 56 were classified as mixed IDC/ILC; for this analyses these patients were categorized as “non-ILC.” We excluded the 55 cases with unknown histology, leaving 5106 patients for analysis.

### Statistical approach

We compared clinicopathologic characteristics in pure ILC versus non-ILC using *t*-tests or Fisher’s exact test for continuous and categorical variables respectively. Within each histologic subtype (ILC versus non-ILC), we further stratified by tumor receptor status (HR+HER2- disease, and non-HR+HER2- disease, which comprised triple negative [HR-HER2-] and HER2+ cases).

The primary endpoint was event free survival (EFS), with any locoregional recurrence, local progression on NAC that precludes surgical resection, distant recurrence, or death from any cause considered an event. Patients without events were censored at the date of last follow up, with follow up time calculated from the start date of NAC. We used univariable and multivariable mixed effects Cox models to evaluate the relationship between continuous RCB index and EFS by histologic subtype overall (ILC and non-ILC) and stratified by receptor subtype. The multivariable model included age at diagnosis, clinical T stage, clinical nodal status, and tumor grade. We plotted the log relative hazard rate for EFS as a function of continuous RCB for ILC and non-ILC cases overall and by receptor subtype. Splines approximation of RCB with two degrees of freedom was used to allow non-linear effect. We calculated estimated cumulative EFS at 3, 5, and 10 years by histologic subtype and stratified by receptor status for each RCB class. Results are reported as hazard ratios (HR) with 95% confidence intervals (CI), with two-tailed *p*-values of <0.05 considered statistically significant.

Finally, recursive partitioning was used in an exploratory model to stratify prognosis by individual clinicopathologic variables and the components of RCB in ILC cases stratified by receptor subtype. Using the *rpart* package in R software, we constructed a survival tree to best predict 10-year EFS. Input variables for the model included clinical T stage, clinical nodal status (positive or negative), estrogen receptor and progesterone receptor status (positive or negative), HER2 receptor status (amplified or non-amplified), tumor grade (1, 2, or 3), patient age (grouped as ≤50 years, 51–70 years, and >70 years) as well as the components of the RCB calculation: primary tumor bed size, invasive cancer cellularity, number of positive lymph nodes, and the size of the largest lymph node metastasis. The tree was pruned based on cross-validation error; the final tree was selected by expanding the tree that minimized cross-validation error to include one additional level (of complexity).

## Results

### Study cohorts and RCB distribution by histology

Of 5106 patients analyzed, 216 (4.2%) had pure ILC, and the remaining 4890 (95.8%) had non-lobular histology (Supplementary Fig. 1). Patients with ILC were diagnosed at older ages compared to those with non-ILC histology (median age 51 years versus 49 years, *p* < 0.001, Table 1). Additionally, the ILC cohort had significantly higher T stage at diagnosis (*p* < 0.001, e.g., 36.6% T3 tumors versus 19.2% T3 tumors in ILC and non-ILC cohorts respectively), a different distribution of tumor grade (e.g., grade 3 in 13.4% versus 59.2% respectively, *p* < 0.001), and a different distribution of receptor subtypes (*p* < 0.001). Among ILC cases, 159 (73.6%) were HR+HER2- compared to 1773 (36.3%) in non-ILC cases. When restricted to HR+HER2- subtypes, the ILC group was still significantly older, had more T3 tumors, and was less likely to have grade 3 tumors compared to the non-ILC group (Table 2). Surgical procedure performed was mastectomy in 53.7% of the ILC group, and 38.7% of the non-ILC group. There was no difference in median follow up time between the ILC and non-ILC cases (median 54 months versus 56 months respectively).

**Table 1:** Clinicopathologic features comparing ILC and non-ILC cohorts (ILC, invasive lobular carcinoma; IQR, interquartile range; HR, hormone receptor; HER2, human epidermal growth factor-2)

		All cases (*n* = 5106)	ILC (*n* = 216)	Non-ILC (*n* = 4890)	*p*-value
Median Age, years (IQR)		49 (42–57)	51 (46–59)	49 (41–57)	**<0.001**
Clinical T category					**<0.001**
	0	11 (0.2%)	1 (0.5%)	10 (0.2%)	
	1	452 (8.9%)	13 (6.0%)	439 (9.0%)	
	2	3109 (60.9%)	102 (47.2%)	3007 (61.5%)	
	3	1017 (19.9%)	79 (36.6%)	938 (19.2%)	
	4	340 (6.7%)	11 (5.1%)	329 (6.7%)	
	Missing	177 (3.5%)	10 (4.6%)	167 (3.4%)	
Clinically node positive		2753 (53.9%)	107 (49.5%)	2646 (54.1%)	0.209
	Missing	179 (3.5%)	8 (3.7%)	171 (3.5%)	
Histological grade					**<0.001**
	1	129 (2.5%)	30 (13.9%)	99 (2.0%)	
	2	1677 (32.8%)	127 (58.8%)	1550 (31.7%)	
	3	2922 (57.2%)	29 (13.4%)	2893 (59.2%)	
	Missing	378 (7.4%)	30 (13.9%)	348 (7.1%)	
Receptor subtype					**<0.001**
	HR+HER2-	1932 (37.8%)	159 (73.6%)	1773 (36.3%)	
	HR+HER2+	855 (16.7%)	28 (13.0%)	827 (16.9%)	
	HR-HER2-	1751 (34.3%)	19 (8.8%)	1732 (35.4%)	
	HR-HER2+	568 (11.1%)	10 (4.6%)	558 (11.4%)	
Follow-up information					
	Median follow-up, months (IQR)	56 (35–96)	54 (40-100)	56 (35-95)	
	Event-free survival events	1148	56	1092	

Bold indicates statitistical significant (*p* < 0.05).

**Table 2:** Clinicopathologic features comparing ILC and non-ILC cohorts stratified by receptor subtype (ILC, invasive lobular carcinoma; IQR, interquartile range; HR, hormone receptor; HER2, human epidermal growth factor-2; TN, triple negative)

		HR+HER2- ILC (*n* = 159)	HR+HER2-non-ILC (*n* = 1773)	*p*-value	TN or HER2+ILC (*n* = 57)	TN or HER2+ non-ILC (*n* = 3117)	*p*-value
Median age, years (IQR)		50 (46–59)	49 (42–57)	**<0.001**	52 (46–61)	49 (41–57)	**0.015**
Clinical T category				**<0.001**			0.161
	0	1 (0.6%)	4 (0.2%)		0 (0.0%)	6 (0.2%)	
	1	9 (5.7%)	136 (7.7%)		4 (7.0%)	303 (9.7%)	
	2	72 (45.3%)	1110 (62.6%)		30 (52.6%)	1897 (60.9%)	
	3	61 (38.4%)	343 (19.3%)		18 (31.2%)	595 (19.1%)	
	4	8 (5.0%)	113 (6.4%)		3 (5.2%)	216 (6.9%)	
	Missing	8 (5.0%)	67 (3.8%)		2 (3.5%)	100 (3.2%)	
Clinically node positive		78 (49.1%)	1023 (57.7%)	**0.037**	29 (50.9%)	1623 (52.1%)	0.894
	Missing	6 (3.8%)	59 (3.3%)		2 (3.5%)	112 (3.6%)	
Histological grade				**<0.001**			**<0.001**
	1	24 (15.1%)	79 (4.5%)		6 (10.5%)	20 (0.6%)	
	2	99 (62.3%)	805 (45.4%)		28 (49.1%)	745 (23.9%)	
	3	10 (6.3%)	763 (43.0%)		19 (33.3%)	2130 (68.3%)	
	Missing	26 (16.4%)	126 (7.1%)		4 (7.0%)	222 (7.1%)	

Bold indicates statistical significance (*p* < 0.05).

**Fig. 1:**
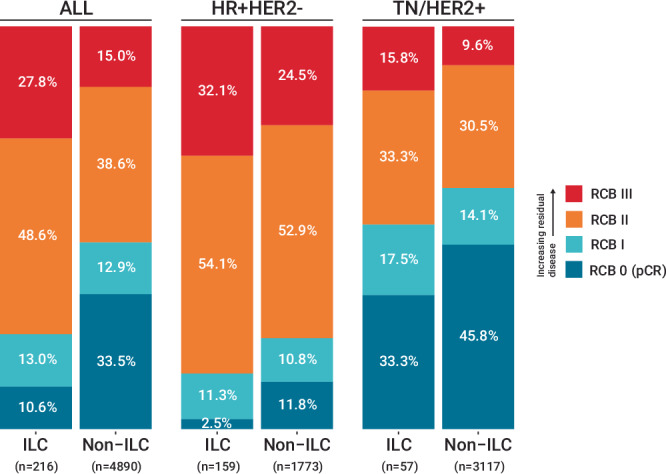
RCB class distribution in ILC vs. Non-ILC cases, overall and by receptor subtype. The distribution of RCB class differs significantly by histologic group overall and among HR+HER2- cases (*p* < 0.001 for both, Fisher’s exact test), but not among TN or HER2+ cases (*p* = 0.163, Fisher’s exact test). (ILC invasive lobular carcinoma, HR hormone receptor, HER2 human epidermal growth factor-2, TN triple negative).

The distribution of RCB class differed significantly by histologic subtype (e.g., 10.6% RCB 0 disease in the ILC cohort compared to 33.5% in the non-ILC cohort, and 27.8% RCB III disease compared to 15.0% RCB III in the ILC and non-ILC cohorts respectively, *p* < 0.001) (Fig. 1). Among HR+HER2- cases only, differences were still found in the RCB distribution (*p* < 0.001). Those with ILC still had lower rates of RCB 0 disease than non-ILC cases (2.5% versus 11.8%, respectively) and higher rates of RCB III disease (32.1% versus 24.5%, respectively). In the subset of ILC cases with triple negative or HER2 amplified tumors (*n* = 57), RCB 0 rates were higher, but still below that of the non-ILC triple negative or HER2 positive cases (33.3% versus 45.8%, respectively, *p* = 0.062 for overall RCB class rates), while RCB III class remained more common among the ILC cohort (15.8% versus 9.6% in non-ILC). Of note, all RCB classes were observed among ILC patients regardless of clinical T stage and clinical nodal status (Supplementary Table 2).

### Associations between RCB and EFS in ILC and non-ILC cohorts

**Fig. 2:**
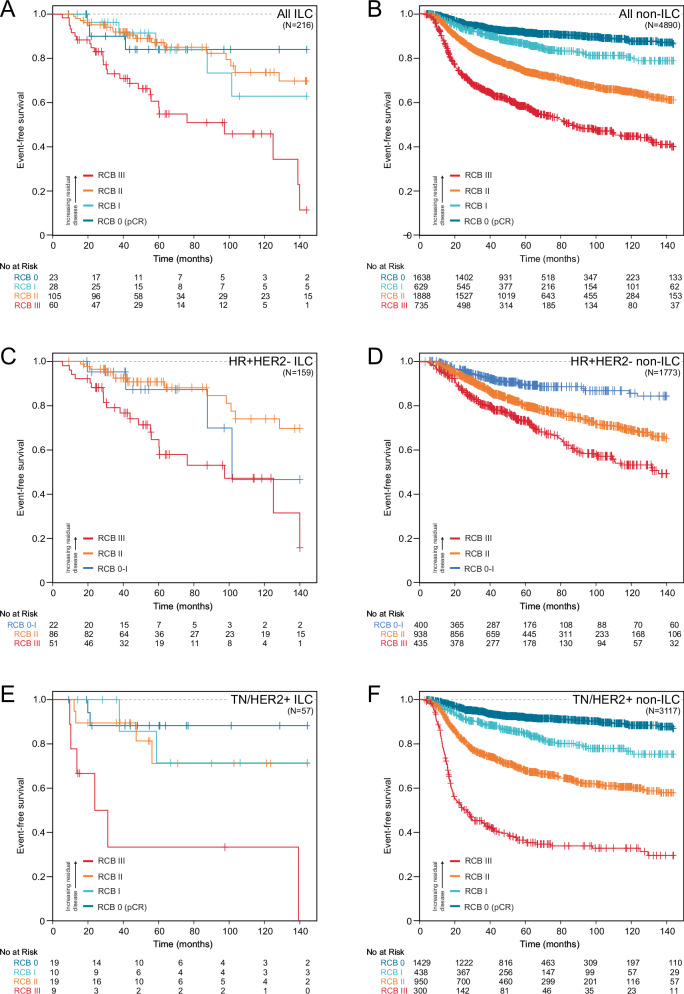
Kaplan–Meier curves for EFS by RCB class in ILC vs. Non-ILC Across Receptor Subtypes. By the log-rank test, there is a significant association between RCB class and EFS for all ILC (**A**) and non-ILC (**B**) cases, HR+HER2- ILC (**C**) and non-ILC (**D**) cases, and TN or HER2+ILC (**E**) and non-ILC (**F**) cases; *p* < 0.001 for all. Of note, for HR+HER2- groups, RCB 0 and RCB I cases were combined due to only 4 ILC cases having RCB 0 disease. Number at risk shown. (ILC invasive lobular carcinoma, HR hormone receptor, HER2 human epidermal growth factor-2, TN triple negative).

Overall, median follow-up time in the study cohort was 56 months (range 35–96), with 1148 EFS events (Table 1). Of these, distant recurrence comprised the majority of events (78.5%), followed by death and local recurrence (13.6% and 7.8%, respectively). Increasing RCB index was associated with significantly shorter EFS in the pure ILC cohort (log relative hazard ratio [HR] per 1 unit increase in RCB compared to RCB 0 was 1.62, 95% CI 1.20–2.19, *p* = 0.002, Table 3). This was true among both the HR+HER2- ILC cases (HR 1.46, 95% CI 1.02–2.11, *p* = 0.041), and among the triple negative or HER2 positive ILC cases (HR 2.21, 95% CI 1.15–4.22, *p* = 0.017). In a multivariable model including age at diagnosis, T stage, clinical nodal status, and tumor grade, RCB index remained significantly associated with shorter EFS among the ILC cohort overall (HR 1.82, 95% CI 1.23–2.68, *p* = 0.003), but in the smaller subset of HR+HER2- ILC cases the trend towards shorter EFS persisted but no longer reached statistical significance (HR 1.53, 95% CI 1.0–2.44, *p* = 0.077) (Table 4). Similarly, for the 57 triple negative or HER2+ILC cases, RCB index was significantly associated with EFS on univariate analysis (Table 3) but failed to reach significance within the multivariate analysis (Table 4). RCB class was significantly associated with EFS on univariate analyses of ILC cases (*p* < 0.0001) with those with RCB III disease having notably shorter EFS than those with RCB 0, I, or II status, which was true overall and among all receptor subtype groups (Fig. 2)

**Table 3:** Estimated cumulative event free survival (EFS) at 3, 5, and 10 years, and univariable associations between RCB index and class with EFS (hazard ratios with 95% confidence intervals and associated *p*-values shown); data shown by histologic group and stratified by receptor subtype

	*N* (%)	EFS at 3 years	EFS at 5 years	EFS at 10 years	Hazard Ratio (95% CI)	*p*-value
ILC (*n* = 216)						
RCB index (continuous)					1.62 (1.20–2.19)	**0.002**
RCB class						
RCB 0	23 (10.6%)	90.0% (77.8–100.0%)	84.0% (68.8–100.0%)	84.0% (68.8–100.0%)	REF	N/A
RCB I	28 (13.0%)	96.3% (89.4–100.0%)	83.9% (67.9–100.0%)	62.9% (40.0–98.9%)	1.75 (0.42–7.35)	0.440
RCB II	105 (48.6%)	91.9% (86.7–97.5%)	87.2% (80.1–94.9%)	73.7% (62.1–87.4%)	1.72 (0.42–7.10)	0.450
RCB III	60 (27.8%)	73.0% (62.2–85.8)	60.7% (48.1–76.4%)	45.9% (31.7–66.4%)	6.53 (1.60–26.6)	**0.009**
Non-ILC (*n* = 4890)						
RCB index (continuous)					1.82 (1.73–1.91)	**<0.001**
RCB class						
RCB 0	1638 (33.4%)	94.1% (93.0–95.3%)	91.4% (89.9–92.9%)	87.6% (85.1–90.1%)	REF	N/A
RCB I	629 (12.8%)	90.9% (88.6–93.2%)	86.3% (83.4–893%)	80.4% (76.3–84.7%)	1.96 (1.51–2.54)	**<0.001**
RCB II	1888 (38.7%)	81.2% (80.2–83.8%)	73.8% (71.7–76.0%)	64.8% (62.1–67.7%)	4.04 (3.33–4.91)	**<0.001**
RCB III	735 (15.1%)	65.9% (62.4–69.5%)	58.1% (54.4–62.1%)	44.7% (40.2–49.7%)	8.93 (7.24–11.01)	**<0.001**
HR+HER2- ILC (*n* = 159)						
RCB index (continuous)					1.46 (1.02–2.11)	**0.041**
RCB class						
RCB 0/I	22 (13.8%)	95.2% (86.6–100.0%)	87.3% (71.8–100.0%)	46.6% (18.3–100.0%)	REF	N/A
RCB II	86 (54.1%)	92.4% (86.8–98.5%)	90.7% (84.4–97.6%)	74.0% (60.9–89.9%)	1.76 (0.23–4.03)	0.390
RCB III	51 (32.1%)	79.0% (68.2–91.6%)	64.6% (51.2–81.5%)	47.1% (31.6–70.3%)	2.35 (0.86–6.41)	0.096
HR+HER2- non-ILC (*n* = 1773)						
RCB index (continuous)					1.54 (1.38–1.71)	**<0.001**
RCB class						
RCB 0/I	400 (22.6%)	93.3% (90.8–95.9%)	89.2% (85.8–92.7%)	85.5% (80.8–90.5%)	REF	N/A
RCB II	938 (52.9%)	88.5% (86.4–90.6)	79.8% (77.0–82.7%)	69.2% (65.3–73.3%)	1.97 (1.44–2.69)	**<0.001**
RCB III	435 (24.5%)	80.5% (76.8–84.5%)	72.9% (68.5–77.7%)	52.2% (47.0–60.2%)	3.38 (2.44–469)	**<0.001**
TN or HER2+ILC (*n* = 57)						
RCB index (continuous)					2.21 (1.15–4.22)	**0.017**
RCB class						
RCB 0	19 (33.3%)	88.2% (74.2–100.0%)	88.2% (74.2–100.0%)	88.2% (74.2–100.0%)	REF	N/A
RCB I	10 (17.5%)	100.0% (100.0–100.0%)	71.4% (44.7–100.0%)	71.4% (44.7–100.0%)	1.36 (0.15–12.10)	0.790
RCB II	19 (33.3%)	89.5% (76.7–100.0%)	71.2% (49.8–100.0%)	71.2% (49.8–100.0%)	5.37 (0.64–45.16)	0.120
RCB III	9 (15.8%)	33.3% (11.3–98.5%)	33.3% (11.3–98.5%)	33.3% (11.3–98.5%)	26.87 (2.59–278.94)	**0.006**
TN or HER2+ non-ILC (*n* = 3117)						
RCB index (continuous)					1.91 (1.81–2.02)	**<0.001**
RCB class						
RCB 0	1429 (45.8%)	94.5% (93.3–95.8%)	91.8% (90.2–93.4%)	88.4% (85.9–91.0%)	REF	N/A
RCB I	438 (14.0%)	89.0% (86.0–92.1%)	84.6% (81.0–88.4%)	76.7% (71.4–82.4%)	2.15 (1.61–2.90)	**<0.001**
RCB II	950 (30.6%)	75.4% (72.5–78.3%)	67.8% (64.6–71.1%)	60.6% (56.8–64.6%)	4.34 (3.49–5.40)	**<0.001**
RCB III	300 (9.7%)	44.5% (39.0–50.6%)	36.5% (31.1–42.9%)	32.9% (27.2–39.6%)	11.88 (9.37–15.06)	**<0.001**

In these unadjusted analyses, RCB index is significantly associated with EFS for all subgroups (*p* < 0.05). Note that for the HR+HER2- cases, RCB 0 and RCB I cases were combined as RCB 0/I for class comparisons due to only 4 ILC patients having RCB 0 disease; additionally, EFS estimates at 10 years represent small sample sizes in some subsets (number at risk for all subsets shown in Fig. 2).

Bold indicates statistical significance (*p* < 0.05).

**Table 4:** Multivariable models showing the association between RCB index and class (hazard ratios with 95% confidence intervals and associated *p* values shown), stratified by histologic group and receptor subtype; models include age, T category, nodal status, and tumor grade

	ILC (*n* = 181)	Non-ILC (*n* = 4397)	HR+HER2-ILC (*n* = 129)	HR+HER2- non-ILC (*n* = 1586)	TN or HER2+ILC (*n* = 52)	TN or HER2+ non-ILC (*n* = 2811)
**Multivariate model evaluating RCB index**						
RCB	1.82 (1.23–2.68), ***p*** = **0.003**	1.74 (1.64–1.85), ***p*** < **0.001**	1.53 (1.00–2.44), *p* = 0.077	1.48 (1.31–1.67), ***p*** < **0.001**	4.75 (0.81–27.79), *p* = 0.083	1.83 (1.71–1.96), ***p*** < **0.001**
Age	0.98 (0.95–1.02), *p* = 0.310	1.00 (0.99–1.00), *p* = 0.100	0.99 (0.95–1.03), *p* = 0.720	1.00 (0.99–1.01), *p* = 0.670	0.90 (0.78–1.04), *p* = 0.160	0.99 (0.99–1.00), *p* = 0.110
*T category (reference: T2)*						
T0-1	1.54 (0.45–5.27), *p* = 0.490	1.07 (0.84–1.38), *p* = 0.570	1.31 (0.34–5.01), *p* = 0.690	0.98 (0.67–1.45), *p* = 0.930	2.91 (0.01–728.34), *p* = 0.710	1.15 (0.84–1.59), *p* = 0.380
T3	0.80 (0.23–1.51), *p* = 0.490	1.36 (1.16–1.59), ***p*** < **0.001**	0.77 (0.38–1.56), *p* = 0.460	1.11 (0.85–1.44), *p* = 0.460	1.56 (0.18–13.31), *p* = 0.680	1.47 (1.21–1.78), ***p*** < **0.001**
T4	0.84 (0.18–3.84), *p* = 0.820	1.95 (1.60–2.39), ***p*** < **0.001**	0.54 (0.07–4.43), *p* = 0.560	2.26 (1.63–3.13), ***p*** < **0.001**	3.15 (0.03–288.43), *p* = 0.620	1.82 (1.41–2.36), ***p*** < **0.001**
*Nodal status (reference: node negative)*						
Node positive	1.29 (0.67–2.47), *p* = 0.440	1.19 (1.03–1.37), ***p*** = **0.019**	1.05 (0.50–2.19), *p* = 0.900	1.41 (1.11–1.80), ***p*** = **0.006**	9.06 (0.36–228.13), *p* = 0.180	1.11 (0.93–1.33), *p* = 0.250
*Grade (reference: grade 1 or 2)*						
Grade 3	3.16 (1.37–7.28), ***p*** = **0.007**	1.17 (1.02–1.35), ***p*** = **0.003**	3.01 (1.10–8.26), ***p*** = **0.032**	1.52 (1.23–1.87), ***p*** < **0.001**	3.70 (0.10–143.58), *p* = 0.480	0.88 (0.74–1.07), *p* = 0.200
**Multivariable model evaluating RCB class**						
RCB 0	REF	REF	REF (combined RCB 0/I group)	REF (combined RCB 0/I group)	REF	REF
RCB I	1.43 (0.27–7.62), *p* = 0.680	1.91 (1.46–2.52), ***p*** < **0.001**	REF (combined RCB 0/I group)	REF (combined RCB 0/I group)	0.43 (0.01–28.50), *p* = 0.690	2.08 (1.53–2.83), ***p*** < **0.001**
RCB II	1.45 (0.30–6.95), *p* = 0.640	3.78 (3.08–4.64), ***p*** < **0.001**	0.55 (0.17–1/73), *p* = 0.300	1.99 (1.42–2.77), ***p*** < **0.001**	3.10 (0.06–157.98), *p* = 0.570	4.04 (3.21–5.09), ***p*** < **0.001**
RCB III	6.89 (1.41–33.64), ***p*** = **0.017**	7.38 (5.89–9.24), ***p*** < **0.001**	2.16 (0.64–7.31), *p* = 0.220	3.16 (2.19–4.54),***p*** < **0.001**	222.83 (1.12–44,368.31), ***p*** = **0.045**	9.77 (7.55–12.65), ***p*** < **0.001**
Age	0.97 (0.94–1.01), *p* = 0.130	1.00 (0.99–1.00), *p* = 0.160	0.98 (0.94–1.02), *p* = 0.360	1.00 (0.99–1.01), *p* = 0.800	0.86 (0.71–1.05), *p* = 0.140	0.99 (0.99–1.00), *p* = 0.110
*T category (reference: T2)*						
T0-1	1.50 (0.43–5.18), *p* = 0.520	1.05 (0.82–1.35), *p* = 0.670	0.70 (0.19–2.58), *p* = 0.590	0.93 (0.63–1.38), *p* = 0.730	2.17 (9.67 × 10^–6^–488,756.31), *p* = 0.900	1.12 (0.81–1.55), *p* = 0.480
T3	1.00 (0.54–1.85), *p* = 1	1.40 (1.20–1.64), ***p*** < **0.001**	0.60 (0.16-2.24), *p* = 0.440	1.12 (0.86-1.45), *p* = 0.420	2.80 (0.23–34.37), *p* = 0.420	1.52 (1.25–1.84), ***p*** < **0.001**
T4	1.76 (0.39–8.01), *p* = 0.460	2.02 (1.65–2.48), ***p*** < **0.001**	0.53 (0.05–5.49), *p* = 0.590	2.39 (1.72–3.31), ***p*** < **0.001**	1.90 (0.03–126.25), p = 0.760	1.83 (1.41–2.38), ***p*** < **0.001**
*Nodal Status (reference: node negative)*						
Node positive	1.33 (0.70–2.54), *p* = 0.380	1.36 (1.18–1.57), ***p*** < **0.001**	1.13 (0.56–2.28), *p* = 0.730	1.57 (1.24–1.99), ***p*** < **0.001**	12.83 (0.18–891.08), *p* = 0.240	1.24 (1.04–1.49), ***p*** = **0.018**
*Grade (reference: grade 1 or 2)*						
Grade 3	2.60 (1.14-5.94), ***p*** = **0.023**	1.15 (0.99–1.32), *p* = 0.063	3.05 (1.12–8.35), ***p*** = **0.030**	1.45 (1.18–1.79), ***p*** = **0.001**	5.85 (0.18–193.67), *p* = 0.320	0.87 (0.72–1.05), *p* = 0.140

In these adjusted analyses, RCB index is significantly associated with EFS for ILC and non-ILC cases overall. When stratified by receptor subtype, higher RCB index remains significantly associated with EFS in non-ILC cases, and trends towards shorter EFS in ILC subsets but has wide confidence intervals in these smaller groups. For models including RCB class, RCB 0 and RCB I cases were combined as a single reference group (RCB 0/I) for HR+HER2- cases due to only 4 ILC patients having RCB 0 disease.

Bold indicates statistical significance (*p* < 0.05).

Within the non-ILC cohort, RCB index was significantly associated with shorter EFS overall, and stratified by receptor subtype, with HR 1.82 overall (95% CI 1.73–1.91, *p* < 0.001), HR 1.54 in the HR+HER2- non-ILC cases (95% CI 1.38–1.71, *p* < 0.001), and HR 1.91 in the triple negative or HER2 positive non-ILC cases (95% CI 1.81–2.02, *p* < 0.001) (Table 3). In the multivariable model, increasing RCB index was associated with increased risk of recurrence (HR 1.74, 95% CI 1.64–1.85, *p* < 0.001) in all non-ILC cases, and among the HR+HER2- non-ILC cases (HR 1.48, 95% CI 1.31–1.67, *p* < 0.001) (Table 4). As expected, RCB class was significantly associated with EFS in non-ILC cases across all receptor subtype groups (*p* < 0.001, Fig. 2).

**Fig. 3:**
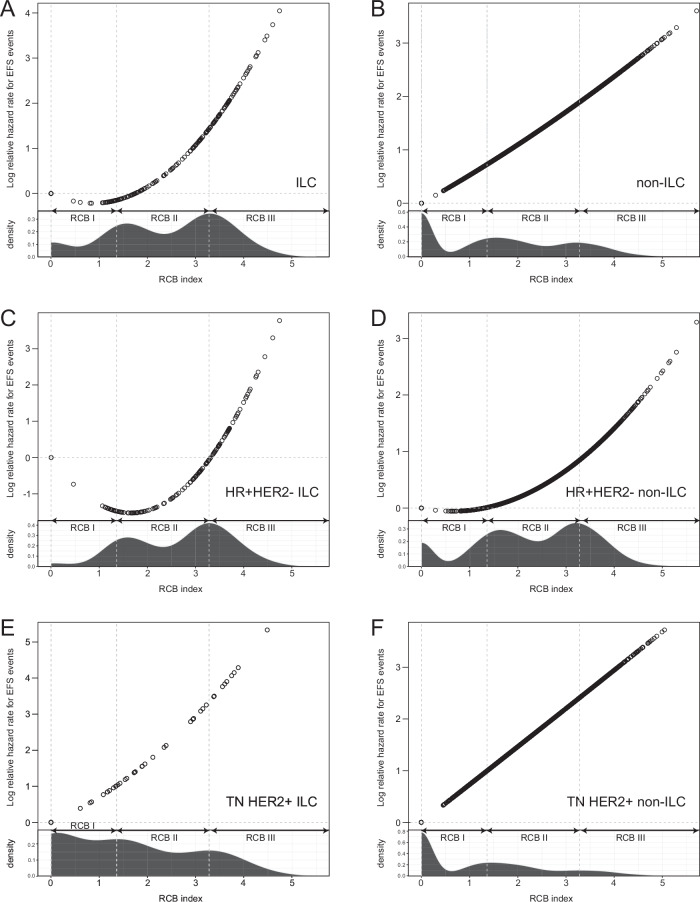
Log relative Hazard Rates for EFS events as a function of RCB Index. All ILC cases shown in (**A**) and non-ILC cases shown in (**B**); **C** shows HR+HER2- ILC, **D** shows HR+HER2- non-ILC, **E** shows TN or HER2+ILC, and **F** shows non-ILC cases; thresholds for corresponding RCB classes (RCB 0 to RCB III) are shown by vertical dashed lines, with RCB index of 0 corresponding to RCB class 0.

While the log-scale relationship between RCB index and increasing risk of EFS event was linear in the non-ILC cohort, this relationship in the ILC cohort was non-linear below RCB index values ≤1.9 (as assessed by visual inspection of plot, Fig. 3A). This non-linear relationship corresponds to similar EFS risk for RCB 0, I, and II cases among the ILC cohort, with significantly shorter EFS for those with RCB III disease (Table 3).

### Estimated cumulative EFS at 3, 5, and 10 years in ILC cases

In the ILC cohort, those with RCB class III disease had significantly shorter estimated EFS compared to RCB class 0 (HR 6.53, *p* = 0.009), with estimated cumulative 3-, 5-, and 10-year survival of 73.0%, 60.7%, and 45.9% for RCB III cases, versus 90.0%, 84.0%, and 84.0% respectively for RCB 0 cases (Table 3). While 10-year EFS was numerically lower for RCB I and RCB II ILC cases compared to RCB 0 cases, this difference was not statistically significant, although at 10-years follow-up time only 36 ILC patients remained for analysis (Table 3 and Fig. 2). When evaluated in only the 159 HR+HER2- ILC cases the relationship between RCB class and EFS was no longer significant (in contrast to RCB index which was associated with EFS in this subset, Table 3). Among the 57 triple negative or HER2+ILC cases, RCB III disease was associated with significantly worse estimated cumulative EFS compared to RCB 0 cases (15.8%, 33.3%, and 33.3% at 3-, 5-, and 10 years versus 88.2% at all timepoints, *p* = 0.006).

### Exploratory recursive partitioning model for EFS

**Fig. 4:**
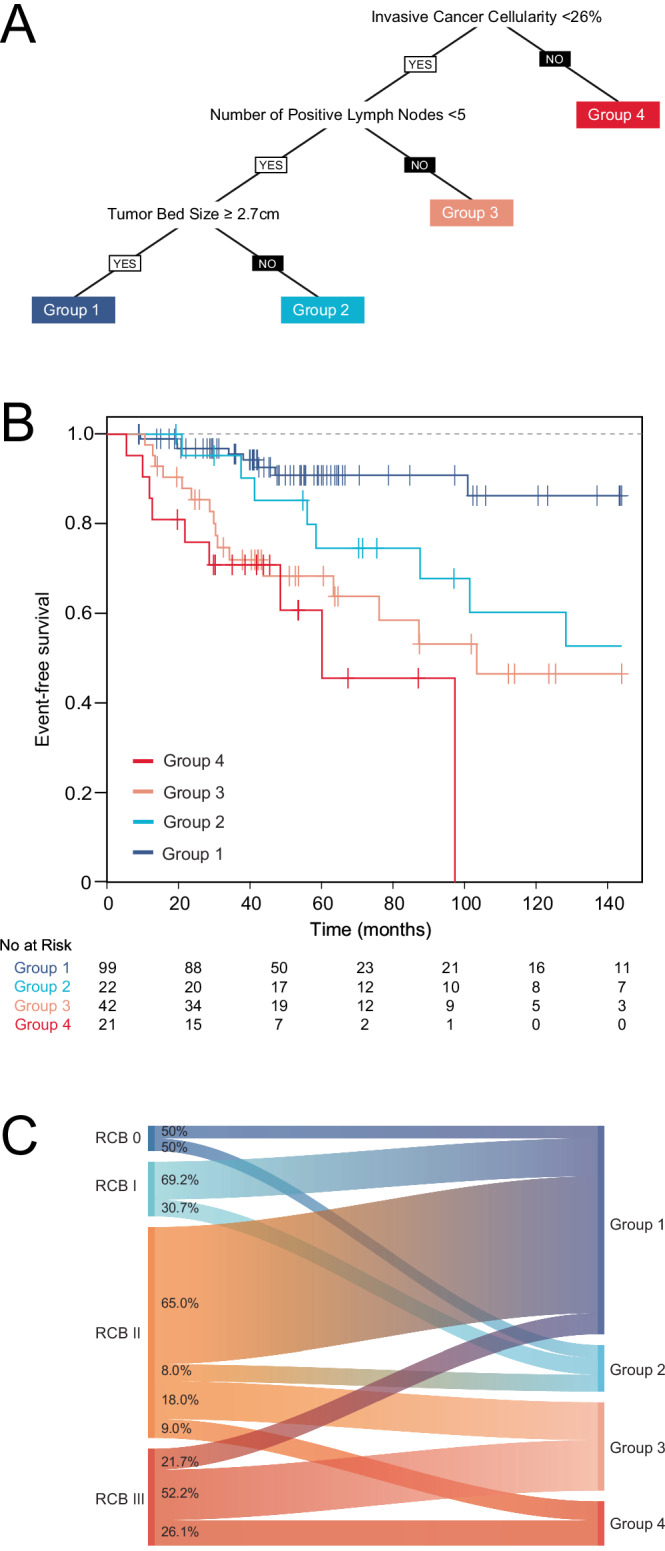
Recursive partitioning model predicting EFS in ILC cases; the model included clinical T category, clinical nodal status, estrogen and progesterone receptor status, HER2 receptor status, tumor grade, age, and all components of RCB calculation (primary tumor bed size, tumor cellularity, percentage in situ carcinoma, number of positive nodes, and size of largest nodal metastasis on surgical pathology). Panel **A** shows 4 distinct group identified by recursive partitioning model with invasive cancer cellularity being first predictor, followed by number of residual positive nodes (5 or more versus <5), followed by primary tumor bed size (with larger size being associated with group 1, and improved EFS). Panel **B** shows Kaplan–Meier Curve for EFS by Recursive Partitioning Groups 1–4, with *p*-value < 0.001 by log rank test (10-year estimated cumulative EFS for group 1 is 86.3% (95% confidence interval 76.2-97.7)). Panel **C** shows the relationship between RCB classes and Recursive Partitioning Groups 1–4.

To better understand which components of the RCB index best predicted EFS in ILC cases, we included individual RCB components along with age and tumor grade in a recursive partitioning model to predict 10-year EFS. In this model, invasive cancer cellularity <26% versus ≥26% first divided the patients into improved versus worse EFS groups respectively (Fig. 4). Among those with tumor cellularity <26%, the next most predictive factor was number of residual positive lymph nodes, with patients having fewer than 5 positive nodes having improved prognosis compared to those with ≥5 positive nodes. Finally, among those with both tumor cellularity <26% and <5 positive nodes, residual tumor diameter next divided the cases. Counterintuitively, those with larger tumors (≥2.7 cm) had improved EFS compared to those with tumor diameter <2.7 cm in size. This recursive partitioning tree identified four distinct groups (1–4) with group 1 having the longest EFS and group 4 having the shortest EFS (Fig. 4). For ILC cases with RCB 0 or RCB I disease, all cases fell into the two best prognostic groups (group 1 or 2) identified by the recursive partitioning tree. In contrast, ILC cases with RCB II or RCB III disease were distributed throughout the recursive partitioning groups 1–4. For RCB II cases, 65% fell into group 1 (best prognosis) while 27% fell into groups 3 or 4 (shortest EFS). For RCB III cases, 21.7% fell into group 1, while 52.2% and 26.1% fell into groups 3 and 4 respectively.

## Discussion

In this study of 216 ILC and 4890 non-ILC cases who received NAC across 12 different institutions, we showed that RCB index and class have significant prognostic capability regardless of histologic subtype of breast cancer. For HR+HER2- cases, the relationship between RCB and EFS was non-linear, which was more pronounced in the ILC cohort. In prior analyses of this dataset, RCB was shown to be prognostic across receptor subtypes, with RCB 0 and RCB I classes having similar prognosis among HR+HER2- cases. In those cases, the log relative HR for RCB was similar up to an RCB index of 1.5^7^. In the current analysis, we found similar results for the ILC cohort, with the exception of finding similar prognosis for ILC cases with RCB index up to 1.9. This is consistent with RCB 0, I, and II classes having comparable EFS outcomes in the ILC cohort. For HR+HER2- ILC cases specifically, which represent the vast majority of ILC tumors seen in clinical practice, estimated cumulative EFS at 5 years was high for those with either RCB 0/I or RCB II disease (87.3% and 90.7% respectively), with a lower 5-year EFS observed in those with RCB III disease (64.6%). These findings suggest that clinical trials utilizing RCB as an endpoint should consider even RCB II status as a good prognostic indicator in patients with HR+HER2- ILC, with the caveat that longer term follow up is needed to exclude an impact on late recurrence.

There are several potential explanations for the non-linear relationship between RCB and EFS in ILC. This finding could reflect underlying biological differences between ILC and IDC, including decreased sensitivity to chemotherapy in ILC (evidenced by a lower rate of RCB 0 and RCB I disease) combined with a differential sensitivity to endocrine therapy. Prior analyses demonstrate that a combination of baseline prognostic features (including clinical stage) along with a gene expression index related to hormonal-related transcription (the SET2,3 assay) is significantly associated with outcomes for HR+HER2- breast cancer treated with NAC^17^. A validated measure, SET2,3 adds prognostic information to RCB with an additive effect, suggesting that increased sensitivity to endocrine therapy can potentially compensate for lesser response to chemotherapy. For those with high sensitivity to chemotherapy, indicated by RCB 0 disease, the risk of recurrence is low, and the addition of adjuvant endocrine therapy may have less impact. However, for those with a low to moderate amount of residual disease, increased or decreased sensitivity to adjuvant endocrine therapy could result in differential risk of recurrence. Notably, high SET2,3 status is less common in basal tumors or molecularly higher-risk tumors, which are under-represented in ILC cases^18–20^. Consequently, the non-linear relationship between RCB index and EFS below RCB score of 1.9 for ILC cases could reflect differences in the distribution of SET2,3 between ILC and IDC cases. Additionally, among RCB III cases, we note numerically lower estimated cumulative EFS at 3, 5, and 10-year time points for the HR+HER2- ILC cases compared to HR+HER2- non-ILC cases; this could reflect differences in baseline prognosis due to clinical stage despite a potentially higher sensitivity to endocrine therapy, and highlights the need for both improved detection and improved systemic therapy specifically for patients with ILC. Alternatively, the small number of ILC cases with RCB 0 or RCB I disease means that even rare EFS events in this group could drive non-linearity in the relationship between RCB and EFS; for example, we note that by 5 years of follow-up time, there are only 7 HR+HER2- ILC cases with RCB 0/I disease, of whom 2 experienced EFS events. These events result in worse than expected estimated cumulative 10-year EFS with wide confidence intervals (46.6%, 95% CI 18.3–100.0%, Table 3), which contributes to the apparent non-linearity between RCB and EFS, but could result from lack of statistical power or type II error.

Of note, recent data suggest that low sensitivity to chemotherapy does not necessarily imply increased sensitivity to endocrine therapy. Analysis of 283 node positive ER-positive patients from SWOG 8814 evaluated the relationship between 21-gene Recurrence Score (RS) and the SET2,3 assay^21^. The proportion of patients with low SET2,3 was similar in those with low versus high 21-gene RS (47% and 51%, respectively). These data suggest that ILC tumors likely vary in both their sensitivity to chemotherapy and their sensitivity to endocrine therapy. This may explain some of our results, where lobular cases with RCB II or RCB III disease were distributed across all 4 prognostic groups in our exploratory recursive partitioning model (Fig. 4).

It is important to acknowledge that the proportion of ILC tumors in this dataset, where only 4.2% of cases had pure lobular histology, is lower than the 10–15% prevalence of ILC observed among all new breast cancer diagnoses. We hypothesize that patients with ILC were less likely to be selected for NAC and are therefore underrepresented in this multi-institutional database. Because several studies show that ILC tumors have lower response rates to NAC, as measured by pCR rates, there is a prevailing belief among some physicians that chemotherapy is less effective in ILC^22^. While this may be true, there is likely a subset of ILC patients who do indeed benefit from chemotherapy, especially since data show molecular heterogeneity within ILC^18,19,23^. Although data on successful breast conservation after NAC are mixed, an analysis of tumor resection volume which included some patients with ILC showed that NAC was associated with significantly lower resection volume compared to adjuvant chemotherapy^24^. Additionally, adjuvant chemotherapy for patients with stage III ILC could confer survival benefit, as stage III patients are typically excluded from trials omitting chemotherapy^20,25^. This question deserves further study and is especially important for patients with ILC since they face significantly higher rates of discordant genomic and clinical risk, likely due to presentation at higher stage resulting from decreased sensitivity of screening mammography^11,12^. Determining whether chemotherapy should be routinely given in the neoadjuvant setting for those with higher stage ILC, despite a tendency towards low-risk genomic scores in this tumor type, is perhaps even more challenging. Given the high rates of ER positivity in ILC, the use of neoadjuvant endocrine therapy may be an alternative strategy that helps identify those patients who might benefit from chemotherapy in the adjuvant setting based on tumor response^26–28^.

While molecular differences between ILC and IDC may contribute to our findings, morphologic differences in tumor growth pattern may also influence RCB assessment. The methodology for the pathologic assessment of residual disease has improved over time, with more recent studies showing a stronger association between the presence of pathologic complete response and EFS; such findings suggest increased accuracy of diagnosis^29^. In our study, reduced precision of RCB assessment in lobular cases could account for the observed decreased linear relationship between RCB and EFS in HR+HER2- ILC. The lack of the adhesion protein E-cadherin results in a diffuse growth pattern with single file lines of tumor cells. The histologic assessment of ILC is known to be especially challenging, with only moderate concordance between pathologists for the diagnosis of ILC in some studies^30,31^. When the histologic pattern of tumor cells after NAC showed scattered residual tumor cells singly or in small islands, measurements of pathologic tumor size is more challenging^32^. Particularly when assessing for small amount of residual disease after NAC, differences in specimen sampling methodology could have a greater impact on ILC cases than IDC cases, rendering the RCB classification less accurate, particularly when the classification is of low or intermediate levels of residual disease such as RCB I or RCB II. Additionally, the higher rates of mastectomy in the ILC group could exacerbate this issue, since identifying residual disease in mastectomy specimens without contemporary methods for localization of the tumor bed may be more challenging^33^.

Alternatively, the morphological differences in the growth pattern of ILC compared to IDC may indeed result in differential patterns of response to therapy. A concentric decrease in tumor size after NAC is less common in patients with HR+HER2- disease, and patients with ILC may be even more likely to have a scattered residual pattern of disease due to its diffuse growth pattern^34^. Investigators have recently shown that the histologic pattern of response is associated with outcomes, such that the presence of scattered residual disease portends increased risk of recurrence relative to tumors with concentric shrinkage^35^. However, this finding was strongest in triple-negative breast cancers, which are rare in ILC. As such, significant chemotherapy response in ILC may be best reflected by reduction in tumor cellularity as opposed to reductions in tumor bed diameter or even nodal positivity. Indeed, our recursive partitioning tree identified tumor cellularity as the most predictive component of RCB for the ILC cases. In this model, we found that among those cases with relatively lower tumor cellularity, the combination of fewer residual positive nodes along with larger tumor size was paradoxically associated with the most favorable prognosis. While this finding could be an anomaly due to small sample size or occasional inaccurate RCB assessment, it is also possible that the combination of a larger tumor without extensive nodal involvement indicates the underlying biology of the tumor, with perhaps a lower potential for metastatic spread despite larger size. While larger tumor size in breast cancer is associated with increased likelihood of nodal involvement, this correlation is weaker at the extremes of tumor size and is impacted by tumor biology, suggesting that larger size alone may not reflect a tumor’s propensity for distant dissemination^36,37^. Prior work shows a relationship between tumor cellularity and pathologic tumor size after NAC, with smaller residual tumors having a larger decrease in cellularity^38^. Whether this association between reduction in cellularity and reduction in tumor size holds true for lobular tumors specifically is unknown, but our findings suggest that lack of reduction in tumor size after NAC may not always be a negative prognostic indicator for those with ILC.

There are several limitations to this analysis, including non-centralized RCB assessment over varying time periods and differences in institutional approaches to the diagnosis of ILC regarding selective or routine E-cadherin staining. Additionally, despite pooling 12 studies, the lower prevalence of ILC, particularly in NAC trials, limits our sample size of ILC cases. Among the HR+HER2- ILC cases, there were only 4 with RCB 0 and 16 with RCB I disease, reducing our statistical power. However, the scant number of ILC cases with RCB 0 disease highlights the importance of developing alternatives to pCR alone as an endpoint for patients with ILC. Finally, HR+HER2- ILC is known for risk of late recurrence, making the median follow-up time of 54 months relatively short.

These findings highlight the need for improved strategies to identify patients with ILC who are predicted to have good response to NAC; while the proportion of patients with RCB 0 or RCB I disease was small, there was a subset of patients with ILC with excellent response to NAC, suggesting a subset with chemotherapy sensitivity. Additionally, we found that the RCB method was associated with EFS in ILC, emphasizing the importance of careful pathologic assessment to provide prognostic information. Our finding of tumor cellularity being the strongest predictor of EFS for ILC cases is intriguing, and validation of the classification system identified by the recursive partitioning model would be of interest. Finally, testing whether a combination of RCB and SET 2,3 would provide even greater prognostic ability for patients with ILC could be particularly useful.

## Supplementary information

Supplemental Material

## Data Availability

De-identified data are subject to European and U.S. privacy laws and may be made available upon request to the corresponding author, with appropriate institutional approvals obtained.

## Code availability

Custom R code used in this analysis is available upon request from the corresponding author.
