# Supplementary material for: A pooled analysis evaluating prognostic significance of Residual Cancer Burden in invasive lobular breast cancer
Source: NPJ Breast Cancer. 2025 Feb 13;11:14. doi: 10.1038/s41523-025-00720-3 (PMC11825822; doi:10.1038/s41523-025-00720-3)
Supplement: Supplementary file 1 — Supplemental Material [file 41523_2025_720_MOESM1_ESM.pdf]

Supplementary Figure 1: Study Cohort Flow Diagram

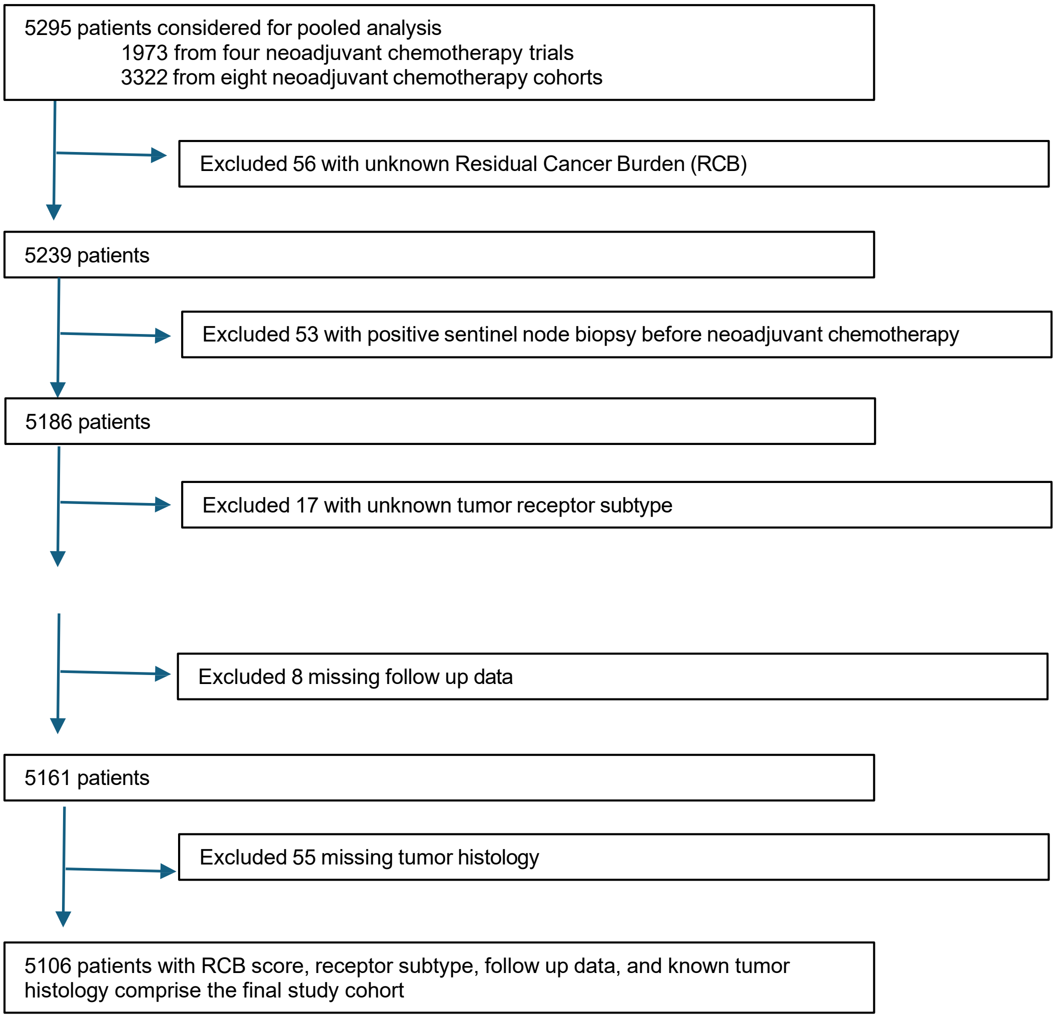

Supplementary Table 1: Ethical approval, type of consent obtained, and treatment period by each contributing cohort

Cohort	Ethical Approval	Type of Consent	Treatment Period
I-SPY 1	IRB at each trial site (ACRIN 6657/CALGB 150012 and CALGB 150007)	Written	06/12/2002 - 03/01/2006
I-SPY 2	IRB at each trial site (NCT01042379)	Written	04/12/2010 - 10/27/2016
MDACC	IRB protocols LAB98-240, LAB02-010	IRB approval provided waiver of individual consent	09/12/1994 - 10/19/2011
Curie	CNIL declaration number 157270	CNIL approval provided waiver of individual consent	01/29/2002 - 05/02/2012
IISGM	Ethnical Board at each participating institution (NCT 01560663)	Written	07/22/2010 - 02/11/2019
KUMC	IRB at each participating institution (NCT02302742)	Written	04/23/2008 - 03/23/2018
TransNeo	REC reference 12/EE/0484	Written	03/15/2013 - 03/04/2017
Yale	IRB Protocol ID 2000025353	Approval to use deidentified information	03/20/2005 - 06/19/2017
Edinburgh	Edinburgh Cancer Information Programme Board reference number CIR21166	Written	09/19/2012 - 08/22/2018
ARTemis	Multi-Centre Research Ethics Committee and research ethics committees at each trial site	Written	05/08/2009 - 01/15/2013
NKI	IRB number IRBd19089/ CFMPB611	Waiver because of deidentified data	12/08/2004 - 03/15/2016
Mayo Clinic	IRB Protocol Number 15-003703	IRB approval provided waiver of individual consent	03/09/2009 - 08/05/2016

Supplementary Table 2: Clinical tumor stage (cT1-4) and clinical nodal status (negative or positive, noted as cN0 or cN+) were available for 206 ILC cases and 151 hormone receptor (HR) positive and HER2 negative ILC cases. All RCB classes were observed regardless of clinical stage (cT and cN status), suggesting that RCB class does not simply reflect extent of pre-treatment disease.

**ILC cases (n=206)**	**RCB 0**	**RCB I**	**RCB II**	**RCB III**
cT1/2, cN0	12 (57%)	11 (41%)	38 (38%)	7 (12%)
cT3/4, cN0	1 (5%)	5 (19%)	17 (17%)	9 (16%)
cT1/2, cN+	5 (24%)	5 (19%)	16 (16%)	22 (39%)
cT3/4, cN+	3 (14%)	6 (22%)	30 (30%)	19 (33%)
**HR+HER2- ILC cases n=151)**	**RCB 0/I**		**RCB II**	**RCB III**
T1/2, cN0	9 (45%)		31 (37%)	6 (12%)
T3/4, cN0	4 (20%)		15 (18%)	9 (19%)
T1/2, cN+	3 (15%)		15 (18%)	18 (38%)
T3/4, cN+	4 (20%)		22 (27%)	15 (31%)
